# Communication in healthcare quality management frameworks: a comparative analysis of international quality systems

**DOI:** 10.1108/IJHCQA-03-2026-0069

**Published:** 2026-06-10

**Authors:** Judit Pripkó

**Affiliations:** Faculty of Health Sciences, Doctoral School of Health Sciences, University of Pécs, Pécs, Hungary

**Keywords:** Healthcare quality management, Quality governance, Communication, Patient safety, Complaint management, Healthcare standards

## Abstract

**Purpose:**

Healthcare quality management frameworks are widely used to strengthen governance, patient safety and organisational accountability in health systems. Although communication is recognised as an important component of safe and effective healthcare, its role within formal quality management frameworks has received limited systematic attention. This study examines how communication is embedded in major international healthcare quality management frameworks and identifies differences in how communication processes are conceptualised across these systems.

**Design/methodology/approach:**

The study applies a comparative framework analysis of six internationally recognised healthcare quality management systems representing different governance models. The analytical unit of the study is the framework rather than individual documents. A qualitative document analysis was conducted using a structured analytical matrix consisting of seven communication-related dimensions: governance communication, patient communication, team communication, complaint communication, information management, communication training and communication in quality improvement.

**Findings:**

Communication appears across all analysed frameworks, although the level of formalisation varies between governance models. Accreditation-based frameworks regulate communication most explicitly, particularly in relation to patient engagement, team coordination and complaint management. In contrast, management standards and policy frameworks tend to integrate communication more indirectly within broader governance and quality improvement processes.

**Research limitations/implications:**

The study focuses on publicly available framework-level standards and guidance documents and does not examine the practical implementation of these frameworks within healthcare organisations. Future research could investigate how communication standards are operationalised in organisational and clinical contexts.

**Practical implications:**

The findings suggest that healthcare leaders selecting or implementing quality management systems should consider the extent to which communication processes are explicitly integrated within the framework.

**Originality/value:**

This study contributes to the literature by providing a comparative analysis of communication integration across international healthcare quality management frameworks and by conceptualising communication as an organisational component of healthcare quality governance.

## 1. Introduction

Improving the quality and safety of healthcare services has become a central priority for health systems worldwide. Healthcare organisations increasingly rely on structured quality management frameworks to strengthen governance, improve patient outcomes and enhance organisational accountability. These frameworks provide systematic mechanisms for monitoring performance, standardising clinical processes and supporting continuous quality improvement in complex healthcare environments (Donabedian, 1988; Øvretveit, 2009; Shaw and Groene, 2010).

The concept of healthcare quality management has evolved significantly over the past several decades. Early theoretical approaches focused primarily on the evaluation of clinical outcomes and professional standards. However, subsequent research highlighted the importance of organisational structures, care processes and governance mechanisms in shaping healthcare quality (Donabedian, 1988). Within this broader perspective, quality management systems aim to establish organisational processes that enable healthcare institutions to deliver safe, effective and patient-centred care while continuously improving their performance (Øvretveit, 2009; Berwick, 2003).

International organisations and accreditation bodies have played an important role in the development of contemporary healthcare quality frameworks. Various systems have emerged to guide healthcare organisations in implementing quality management principles, including accreditation programmes, international management standards and global policy frameworks (Greenfield and Braithwaite, 2008; Shaw *et al*., 2014). Examples include accreditation programmes developed by Joint Commission International and Accreditation Canada, international quality management standards introduced by the International Organization for Standardization, and global policy initiatives promoted by the World Health Organization. In addition, organisations such as International Society for Quality in Health Care contribute to the governance of healthcare quality by establishing international standards for external evaluation systems.

Despite differences in structure and governance models, most healthcare quality frameworks share several common objectives. These include strengthening leadership and accountability, improving patient safety, promoting evidence-based practice and supporting organisational learning (Berwick, 2003; Shaw and Groene, 2010). Within these frameworks, quality management is typically conceptualised as a system of interrelated organisational processes that support the continuous monitoring and improvement of healthcare services.

Communication processes are closely linked to many of these organisational functions. In the context of healthcare quality governance, communication may be understood as the structured exchange, interpretation and transfer of information between healthcare professionals, patients and organisational actors that supports coordination, decision-making, accountability and patient safety. Communication therefore functions not only as an interpersonal activity but also as an organisational mechanism embedded within broader governance and quality improvement processes (Vermeir *et al*., 2015; Kwame and Petrucka, 2021). Effective communication supports coordination among healthcare professionals, facilitates information exchange and contributes to organisational learning within healthcare institutions (Leonard *et al*., 2004; O’Daniel and Rosenstein, 2008). Communication failures have also been identified as a significant contributor to adverse events and patient safety incidents in healthcare settings (Reader *et al*., 2014; Dixon-Woods *et al*., 2012). Recent research has further highlighted the relationship between communication quality, patient safety culture and organisational resilience within healthcare systems (Howick *et al*., 2024; Wiig *et al*., 2020). As a result, communication has increasingly been recognised as an important factor in improving healthcare quality and patient safety.

Nevertheless, communication is rarely conceptualised as an independent dimension of healthcare quality management systems. In most frameworks, communication appears implicitly within broader organisational processes such as leadership, patient engagement, clinical governance and quality improvement mechanisms. As a consequence, the way communication is incorporated into healthcare quality management systems varies considerably across different governance models and institutional contexts.

Although a growing body of literature has examined individual accreditation systems and quality improvement initiatives, relatively limited research has compared multiple international healthcare quality frameworks in order to analyse how communication is integrated within these systems (Greenfield and Braithwaite, 2008; Shaw *et al*., 2014). Comparative analyses of quality governance systems remain relatively scarce, particularly with regard to communication-related organisational processes.

This study seeks to address this gap by examining how communication is incorporated within major international healthcare quality management frameworks. The study applies a comparative framework analysis of six internationally recognised quality systems representing different governance models. By analysing how communication appears within these frameworks, the study aims to contribute to the literature on healthcare quality governance and to provide insights into the relationship between communication processes and quality management systems.

## 2. Literature review

### 2.1 Evolution of healthcare quality management systems

Healthcare quality management has developed into a central component of modern health system governance. Early conceptualisations of healthcare quality focused primarily on clinical performance and professional standards; however, subsequent research emphasised the role of organisational structures and management systems in shaping healthcare outcomes (Donabedian, 1988). Donabedian’s structure–process–outcome model remains one of the most influential frameworks for analysing healthcare quality and has provided the conceptual foundation for many contemporary quality management systems.

During the late twentieth century, healthcare organisations increasingly adopted systematic approaches to quality management influenced by industrial quality management principles such as continuous improvement and organisational learning (Deming, 1986; Juran, 1989). These approaches highlighted the importance of organisational processes, leadership commitment and data-driven decision-making in improving quality outcomes. The adaptation of these principles to healthcare settings led to the development of comprehensive quality management systems that integrate governance structures, clinical processes and monitoring mechanisms (Øvretveit, 2009).

In parallel with these developments, international accreditation systems emerged as key instruments for strengthening healthcare quality governance. Accreditation programmes evaluate healthcare organisations against predefined standards and encourage the implementation of structured quality improvement processes (Greenfield and Braithwaite, 2008; Shaw and Groene, 2010). Empirical studies have shown that accreditation systems can contribute to improvements in organisational performance, patient safety practices and the development of quality management infrastructures within healthcare organisations (Alkhenizan and Shaw, 2011).

More recently, global health organisations have promoted system-level approaches to healthcare quality governance. International initiatives emphasise the integration of quality management principles within broader health system governance structures, including leadership accountability, patient-centred care and continuous quality improvement (WHO, 2018; Shaw *et al*., 2014). These developments have led to the proliferation of international frameworks that guide healthcare organisations in implementing quality management practices.

Despite the growing importance of these frameworks, healthcare quality management systems vary considerably in their governance models, organisational structures and operational mechanisms. Some frameworks operate as accreditation systems evaluating healthcare providers against explicit organisational standards, while others function as management standards or policy-oriented frameworks guiding national health system reforms (Greenfield and Braithwaite, 2008; Shaw *et al*., 2014). Understanding these differences is essential for analysing how quality governance operates across healthcare systems.

### 2.2 Accreditation, governance and organisational quality improvement

Accreditation programmes represent one of the most widely used mechanisms for implementing healthcare quality management systems. Accreditation standards typically address key organisational domains including leadership, patient safety, clinical governance, workforce competence and information management (Shaw and Groene, 2010). Through external evaluation processes, accreditation programmes aim to ensure that healthcare organisations maintain acceptable standards of care while continuously improving their organisational processes.

Research has shown that accreditation systems can influence healthcare organisations in several ways. Accreditation may promote organisational learning, encourage the adoption of evidence-based practices and strengthen leadership engagement in quality improvement initiatives (Greenfield and Braithwaite, 2008). In addition, accreditation standards often require healthcare organisations to establish internal monitoring systems and quality improvement mechanisms, thereby contributing to the institutionalisation of quality management practices (Øvretveit, 2009).

Quality improvement research has also highlighted the importance of organisational culture, leadership engagement and system-level coordination in achieving sustainable improvements in healthcare quality (Berwick, 2003; Bate and Robert, 2007). Quality management frameworks therefore increasingly emphasise governance structures that support organisational learning, feedback mechanisms and continuous improvement cycles (Dixon-Woods *et al*., 2012).

However, the implementation of quality management systems also presents significant organisational challenges. Healthcare organisations operate in complex environments characterised by professional hierarchies, fragmented information flows and high levels of uncertainty (Waring, 2007). Effective quality governance therefore requires coordination across multiple organisational actors, including clinical professionals, managers and patients.

### 2.3 Communication in healthcare quality governance

Communication processes play a critical role in many of the organisational mechanisms that underpin healthcare quality management systems. Effective communication supports coordination between healthcare professionals, facilitates the transfer of clinical information and enables organisations to identify and address quality and safety risks (Leonard *et al*., 2004; O’Daniel and Rosenstein, 2008). Communication failures have repeatedly been identified as a significant contributing factor in adverse events and patient safety incidents (Reader *et al*., 2014).

Communication is also closely linked to organisational learning processes within healthcare organisations. Recent studies have also emphasised the role of communication in strengthening patient-centred organisational cultures and improving healthcare team effectiveness (Sharkiya, 2023; Meneses-La-Riva *et al*., 2025). Feedback systems, incident reporting mechanisms and patient complaint management structures provide channels through which organisations can detect systemic weaknesses and initiate quality improvement actions (Bate and Robert, 2007; Dixon-Woods *et al*., 2012). In this sense, communication functions as a key enabling mechanism for organisational learning and quality improvement.

In addition, communication has become increasingly important in the context of patient-centred care and participatory health governance. Contemporary quality frameworks emphasise the importance of engaging patients and families in decision-making processes and incorporating patient feedback into organisational learning systems (WHO, 2018). Patient complaints and feedback mechanisms may therefore serve as valuable sources of information for identifying quality problems and improving healthcare services (Reader *et al*., 2014).

Within the context of healthcare governance, communication can also be understood as an institutional process that connects organisational accountability, transparency and stakeholder engagement. From this perspective, communication is not merely an interpersonal interaction but an organisational mechanism embedded within broader governance structures.

Despite the recognised importance of communication in healthcare organisations, its role within formal healthcare quality management frameworks remains relatively underexplored. Existing studies have primarily focused on clinical communication or patient–provider interaction, while less attention has been paid to how communication processes are incorporated into organisational quality management systems.

### 2.4 Comparative analysis of healthcare quality frameworks

Although numerous healthcare quality frameworks have been developed internationally, comparative analyses of these systems remain relatively limited in the literature. Most studies examine individual accreditation programmes or national quality initiatives rather than comparing multiple frameworks across different governance models (Shaw *et al*., 2014). However, comparative analyses are essential for understanding how different institutional approaches shape the implementation of healthcare quality management systems.

Different quality frameworks may emphasise distinct organisational priorities and governance mechanisms. Accreditation systems often define explicit operational standards for healthcare providers, whereas management standards and policy frameworks tend to provide broader governance guidance for quality improvement initiatives (Greenfield and Braithwaite, 2008). These differences may influence how key organisational processes—including communication—are conceptualised and regulated within quality management systems.

A comparative framework analysis therefore provides an opportunity to examine how communication appears within different types of healthcare quality governance models. By analysing multiple frameworks simultaneously, it becomes possible to identify common patterns as well as structural differences in how communication is integrated into healthcare quality management systems. Such insights may contribute to a better understanding of the relationship between organisational communication processes and healthcare quality governance.

## 3. Methods

### 3.1 Research design

This study applies a qualitative comparative framework analysis to examine how communication is incorporated within major international healthcare quality management systems. Comparative analysis is widely used in healthcare governance research to identify structural similarities and differences between institutional models of quality management (Shaw *et al*., 2014). In the context of healthcare quality governance, such comparative approaches provide insights into how different organisational and regulatory frameworks conceptualise and operationalise key dimensions of quality management. The methodological approach is informed by qualitative document analysis and qualitative content analysis methodologies commonly applied in health policy and organisational studies (Bowen, 2009; Schreier, 2012).

The analytical unit of this study is the healthcare quality management framework rather than individual documents. This approach enables the examination of quality governance models as coherent institutional systems that define organisational standards, governance mechanisms and quality improvement processes. Analysing frameworks at the system level allows for the identification of broader patterns in how communication processes are embedded within quality management structures.

The study focuses on six internationally recognised healthcare quality frameworks representing different governance models. These include accreditation-based systems, international management standards and policy-oriented quality frameworks. By comparing frameworks representing these distinct governance approaches, the study aims to identify differences in the integration of communication within healthcare quality management systems.

### 3.2 Selection of healthcare quality frameworks

The selection of frameworks followed a purposive sampling strategy commonly used in qualitative comparative research. The study focused on internationally recognised quality management systems that have been widely implemented or referenced in healthcare governance literature (Greenfield and Braithwaite, 2008; Shaw *et al*., 2014).

Six frameworks were selected for analysis:

1. frameworks based on international management standards developed by the International Organization for Standardization
2. accreditation programmes such as those developed by Joint Commission International and Accreditation Canada
3. global quality policy frameworks promoted by the World Health Organization
4. international meta-accreditation standards developed by the International Society for Quality in Health Care
5. national quality and patient safety standards such as the National Safety and Quality Health Service standards developed by the Australian Commission on Safety and Quality in Health Care.

These frameworks represent different institutional approaches to healthcare quality governance and therefore provide an appropriate basis for comparative analysis.

### 3.3 Data sources

The analysis was based on publicly available framework-level standards, guidance documents and official descriptions of the selected healthcare quality systems. Publicly accessible documents were chosen in order to ensure transparency and replicability of the analysis. The study did not focus on individual organisational implementation but rather on the conceptual structure of the frameworks themselves.

Documents describing the governance structure, organisational standards and quality management principles of each framework were examined in order to identify how communication is incorporated within these systems.

### 3.4 Analytical framework

The qualitative analysis was conducted using a structured analytical matrix developed to capture key communication-related dimensions within healthcare quality management systems. The analytical framework was informed by the literature on healthcare quality governance, patient safety and organisational communication processes (Leonard *et al*., 2004; Reader *et al*., 2014).

Seven analytical dimensions were used in the comparison:

1. Governance communication
2. Patient communication
3. Team communication
4. Complaint communication
5. Information management
6. Communication training
7. Communication in quality improvement.

Each framework was analysed according to these dimensions in order to identify whether communication appeared explicitly within the framework’s standards or implicitly within broader organisational processes.

### 3.5 Analytical procedure

The analysis involved a qualitative coding process in which each framework was systematically examined according to the seven analytical dimensions. For each dimension, the presence and level of formalisation of communication-related elements were assessed.

A three-level coding scale was applied:

0 – communication not explicitly addressed
1 – communication implicitly embedded within broader organisational processes
2 – communication explicitly defined through standards or formal requirements.

The coded results were then compared across the six frameworks in order to identify common patterns and structural differences in the integration of communication within healthcare quality management systems.

### 3.6 Rationale for comparative framework analysis

A comparative framework analysis was selected as the methodological approach for this study in order to examine how communication is conceptualised across different institutional models of healthcare quality governance. Healthcare quality management systems differ substantially in their organisational structure, regulatory logic and operational mechanisms. Comparative approaches therefore provide a useful analytical strategy for identifying structural similarities and differences between governance models (Shaw *et al*., 2014).

Rather than focusing on the implementation of quality management systems within individual healthcare organisations, this study analyses the frameworks themselves as institutional governance models that define organisational standards, accountability mechanisms and quality improvement processes. Examining the frameworks at the system level enables a structured comparison of how communication is integrated within healthcare quality governance structures.

Comparative framework analysis has been widely applied in health policy and healthcare governance research to analyse institutional differences between regulatory and organisational models (Greenfield and Braithwaite, 2008). By applying this approach, the study aims to identify how communication appears across different types of quality management systems, including accreditation programmes, management standards and policy frameworks.

### 3.7 Ethical considerations

Ethical approval was not required for this study because the research is based exclusively on the analysis of publicly available documents describing international healthcare quality management frameworks. The study does not involve human participants, patient data or organisationally sensitive information.

All analysed materials consist of publicly accessible standards, guidance documents and official framework descriptions published by international organisations. As the study does not involve primary data collection or the analysis of identifiable personal data, it falls outside the scope of research requiring formal ethical approval according to commonly applied research ethics guidelines for document-based policy analysis.

## 4. Results

### 4.1 Communication across healthcare quality management frameworks

The comparative analysis examined how communication is incorporated within six international healthcare quality management frameworks representing different governance models. The results demonstrate that communication appears in all analysed frameworks, although the degree of formalisation and the organisational contexts in which communication is addressed vary considerably across systems.

Across the analysed frameworks, communication most frequently appears in relation to governance processes, patient engagement and quality improvement mechanisms. However, important differences emerge in the way communication is operationalised within specific standards and organisational requirements.

Accreditation-based frameworks tend to define communication in a more explicit and operational manner. These systems typically include detailed standards related to patient communication, interprofessional coordination and complaint management mechanisms. In contrast, management standards and policy-oriented frameworks tend to integrate communication more implicitly within broader organisational governance and quality improvement processes.

The findings suggest that communication is not typically conceptualised as an independent dimension of healthcare quality management systems. Instead, it is embedded within several organisational domains, including leadership governance, patient-centred care, information management and quality improvement structures.

### 4.2 Governance communication

Communication related to organisational governance appears consistently across all analysed frameworks. Governance communication typically includes mechanisms through which leadership structures ensure transparency, accountability and organisational coordination within healthcare institutions.

Frameworks based on management standards emphasise leadership responsibility for communicating organisational goals, quality policies and strategic priorities throughout the organisation. Similarly, policy-oriented frameworks highlight the importance of communication between healthcare organisations, regulatory bodies and other stakeholders in supporting effective health system governance.

In accreditation-based systems, governance communication is often linked to leadership accountability and organisational oversight. Accreditation standards frequently require healthcare organisations to demonstrate that leadership structures promote transparency, information sharing and organisational learning processes related to healthcare quality and patient safety.

These findings indicate that governance communication represents one of the most consistently integrated communication dimensions across healthcare quality management systems. Regardless of their institutional design, all analysed frameworks recognise the importance of communication in supporting organisational coordination and leadership accountability.

### 4.3 Patient communication

Patient communication also emerges as a central component of healthcare quality management frameworks. Most frameworks emphasise the importance of patient engagement, informed decision-making and patient-centred care as key principles of quality healthcare delivery.

In accreditation-based systems, patient communication is typically operationalised through explicit organisational standards requiring healthcare institutions to ensure that patients receive appropriate information about their treatment options, rights and care processes. These standards often emphasise shared decision-making and the involvement of patients and families in care planning.

Policy-oriented frameworks also highlight patient communication as a fundamental element of people-centred healthcare systems. In these frameworks, patient communication is often linked to broader governance objectives such as patient participation, transparency and accountability within healthcare organisations.

Although patient communication is widely recognised across frameworks, the degree of formalisation varies. Some systems define detailed operational requirements for patient communication processes, whereas others address patient communication primarily as a guiding principle of patient-centred healthcare.

### 4.4 Team communication

Communication among healthcare professionals appears in most frameworks primarily in the context of care coordination and patient safety. Interprofessional communication and information transfer between healthcare providers are widely recognised as essential components of safe clinical practice.

Accreditation frameworks typically include explicit standards addressing communication between members of the healthcare team. These standards frequently focus on clinical handover processes, multidisciplinary coordination and the transfer of critical information during patient care transitions.

In contrast, management standards and policy frameworks tend to address team communication more indirectly, often through broader references to workforce collaboration, organisational coordination and professional responsibility. While these frameworks acknowledge the importance of teamwork in healthcare delivery, they rarely define detailed operational requirements for clinical communication processes.

These differences reflect broader variations in the governance logic of healthcare quality management systems. Accreditation programmes tend to regulate operational processes more explicitly, whereas management standards and policy frameworks emphasise organisational principles and governance structures.

### 4.5 Complaint communication

Communication related to patient complaints and feedback mechanisms appears across most of the analysed frameworks, although the level of formalisation differs substantially between governance models. Complaint communication refers to organisational processes through which healthcare institutions receive, manage and respond to patient concerns, complaints and feedback regarding healthcare services.

In accreditation-based frameworks, complaint communication is typically addressed through explicit organisational standards that require healthcare institutions to establish formal complaint management systems. These systems often include procedures for receiving complaints, documenting patient feedback and ensuring timely responses to concerns raised by patients or their families. Such requirements position complaint communication as an important component of organisational accountability and transparency.

Management standards and policy-oriented frameworks also recognise the importance of patient feedback and complaints as mechanisms for improving healthcare quality. However, these frameworks tend to address complaint communication more indirectly, often within broader categories such as stakeholder engagement, feedback mechanisms or quality improvement processes. As a result, complaint communication is frequently embedded within general organisational governance structures rather than defined as a distinct operational process.

The analysis therefore indicates that while complaint communication is acknowledged across most healthcare quality frameworks, the extent to which it is regulated through explicit organisational standards varies considerably across governance models.

### 4.6 Information management and communication

Information management represents another domain in which communication-related processes appear consistently across healthcare quality frameworks. In the analysed systems, information management typically includes requirements related to documentation, information sharing and the management of clinical and organisational data.

Most frameworks emphasise the importance of structured information systems that support the monitoring and evaluation of healthcare quality. These systems facilitate communication within healthcare organisations by enabling the systematic collection, documentation and dissemination of information relevant to patient care and organisational performance.

Accreditation-based frameworks frequently define explicit requirements related to the management of health information systems, including standards for documentation, data governance and information flow within healthcare organisations. These requirements aim to ensure that healthcare professionals have access to accurate and timely information necessary for effective clinical decision-making.

Management standards and policy frameworks similarly recognise the importance of information management for quality governance. In these frameworks, communication often appears in the context of organisational monitoring systems, reporting mechanisms and data-driven quality improvement processes.

Overall, the analysis suggests that information management functions as a key organisational infrastructure through which communication processes are operationalised within healthcare quality management systems.

### 4.7 Communication training and professional competence

Communication training appears in several frameworks as part of broader workforce competence and professional development requirements. Within healthcare quality management systems, training programmes aim to ensure that healthcare professionals possess the necessary skills and competencies to support safe and effective care delivery.

Accreditation-based frameworks tend to define explicit requirements for staff training and professional education, often including elements related to communication skills, teamwork and patient interaction. These requirements reflect the recognition that effective communication among healthcare professionals and between providers and patients contributes to improved patient safety and quality outcomes.

In management standards and policy frameworks, communication training is typically addressed as part of broader workforce development and competence management processes. These frameworks emphasise the need for healthcare organisations to provide ongoing professional education and training programmes that support organisational learning and quality improvement.

However, in many frameworks communication training is not addressed as an independent organisational domain. Instead, it is incorporated within general competence and workforce development standards, reflecting the integration of communication skills within broader professional competencies required in healthcare practice.

### 4.8 Communication in quality improvement processes

Communication also plays an important role in organisational quality improvement processes. Within healthcare quality management frameworks, communication-related mechanisms often appear in relation to feedback systems, incident reporting and organisational learning processes.

Quality improvement systems typically rely on structured feedback mechanisms that enable healthcare organisations to identify performance gaps, analyse adverse events and implement corrective actions. These processes depend on effective communication channels that allow information about quality and safety issues to be shared across organisational levels.

Accreditation-based frameworks frequently include explicit requirements related to incident reporting, feedback mechanisms and organisational learning systems. These mechanisms facilitate communication between healthcare professionals, management structures and quality improvement teams, thereby supporting continuous improvement processes.

Policy frameworks and management standards similarly emphasise the role of communication in monitoring healthcare performance and supporting organisational learning. Through reporting systems, evaluation mechanisms and quality monitoring processes, communication becomes an essential component of healthcare quality governance.

Taken together, these findings indicate that communication is embedded across multiple domains of healthcare quality management systems. However, the way communication is operationalised differs significantly between frameworks, reflecting variations in governance structures and regulatory approaches to healthcare quality management.

Table 1 summarises the comparative analysis of communication dimensions across the six examined healthcare quality management frameworks.

**Table 1:** Communication dimensions across international healthcare quality management frameworks

Framework	Framework type	Governance communication	Patient communication	Team communication	Complaint communication	Information management	Communication training	Quality improvement communication	Communication integration level
ISO	Management standard	2	2	1	1	2	1	2	Medium
JCI	Accreditation system	2	2	2	2	2	2	2	High
ISQua	Meta-accreditation framework	2	1	1	1	2	1	2	Medium
WHO	Policy framework	2	2	1	1	2	1	2	Medium
Accreditation Canada	Accreditation system	2	2	2	2	2	2	2	High
Australian NSQHS	National safety and quality standard	2	2	2	2	2	2	2	High

**Note(s):** Values indicate the level of formalisation of communication within each framework. 0 = communication not addressed in the framework. 1 = communication implicitly embedded within broader organisational processes or governance mechanisms. 2 = communication explicitly defined through formal standards or organisational requirements

**Source(s):** Author’s comparative framework analysis based on publicly available quality management standards and guidance documents

## 5. Discussion

The seven analytical dimensions applied in this study were not developed as a prescriptive communication framework for healthcare organisations. Rather, they were designed as comparative governance dimensions intended to capture how communication-related organisational processes appear across different healthcare quality management systems. Collectively, these dimensions illustrate that communication functions across multiple organisational levels, including leadership governance, patient engagement, workforce coordination, information management and quality improvement processes.

The findings of this study provide new insights into how communication is incorporated within international healthcare quality management frameworks. While communication appears across all examined systems, the analysis demonstrates that the degree of formalisation and the organisational context in which communication is addressed vary considerably across different governance models. These differences reflect broader variations in the institutional logic of healthcare quality governance and the regulatory mechanisms through which quality management systems operate.

One of the most important findings of the analysis is that accreditation-based frameworks tend to regulate communication more explicitly than other types of quality management systems. In these frameworks, communication appears as a clearly defined organisational requirement linked to patient safety, interprofessional coordination and complaint management processes. This observation is consistent with previous research suggesting that accreditation systems often translate quality management principles into operational standards that directly regulate organisational practices (Braithwaite *et al*., 2010; Greenfield *et al*., 2012). By defining explicit requirements for communication processes, accreditation frameworks aim to ensure that healthcare organisations implement structured mechanisms supporting coordination, transparency and patient engagement.

In contrast, management standards and policy-oriented frameworks tend to integrate communication more implicitly within broader governance structures and quality improvement processes. Rather than defining detailed operational requirements for communication practices, these frameworks typically emphasise leadership responsibility, organisational accountability and the establishment of quality improvement infrastructures. Such approaches reflect the broader governance orientation of these frameworks, which focus on system-level principles rather than operational clinical processes (Bevan and Hood, 2006; Healy, 2011).

The results also highlight the central role of communication in organisational learning and quality improvement systems. Communication processes support the flow of information necessary for monitoring performance, analysing adverse events and implementing improvement initiatives. Previous research has emphasised that effective communication channels are essential for developing learning-oriented healthcare organisations capable of identifying systemic problems and responding to quality and safety risks (Edmondson, 2004; Tucker and Edmondson, 2003). Within quality management systems, communication therefore functions as a key enabling mechanism for organisational learning and continuous improvement.

Another important finding relates to the role of complaint communication within healthcare quality governance. The analysis demonstrates that complaint management mechanisms are explicitly regulated in accreditation frameworks, while they are addressed more indirectly in policy-oriented or management-based systems. This difference reflects the growing recognition that patient complaints and feedback can serve as valuable sources of information for identifying quality problems within healthcare organisations (Friele and Sluijs, 2006; Gallagher *et al*., 2003). Complaint communication mechanisms provide an institutionalised channel through which patients can report concerns about care quality, thereby contributing to organisational accountability and quality improvement.

From a governance perspective, the findings also suggest that communication plays an important role in mediating relationships between healthcare organisations, professionals and patients. Contemporary quality management frameworks increasingly emphasise transparency, stakeholder engagement and participatory approaches to healthcare governance (Saltman *et al*., 2011; Walshe and Smith, 2016). Communication processes facilitate these governance mechanisms by enabling the exchange of information between organisational actors and supporting the implementation of accountability structures. Contemporary healthcare governance research increasingly recognises communication as a strategic organisational capability linked to resilience, patient trust and system responsiveness (Wiig *et al*., 2020; Sharkiya, 2023).

These results also contribute to the emerging literature examining the institutional dimensions of healthcare communication. Rather than viewing communication solely as an interpersonal interaction between healthcare professionals and patients, institutional perspectives highlight the organisational and governance functions of communication within healthcare systems. Communication processes play a fundamental role in health promotion, patient engagement and organisational coordination within healthcare systems, as effective information exchange is a core component of modern healthcare governance (Boncz *et al*., 2022).

Within this perspective, complaint communication and patient feedback systems represent important institutional mechanisms linking patient experience with organisational learning processes. Patient complaints can function as important indicators of communication failures and organisational quality problems within healthcare systems (Reader *et al*., 2014; Gallagher *et al*., 2003). Empirical research analysing healthcare complaints has also highlighted the ethical and organisational dimensions of complaint communication in healthcare institutions (Pripkó, 2025). Integrating communication more explicitly into healthcare quality management systems may therefore contribute to strengthening organisational transparency and improving patient trust.

The comparative analysis presented in this study therefore highlights the need to consider communication as an organisational dimension of healthcare quality governance rather than merely a clinical interaction process. By examining how communication appears across different quality frameworks, the study demonstrates that communication functions as an underlying organisational infrastructure supporting governance, coordination and quality improvement processes.

At the same time, the findings suggest that the integration of communication within healthcare quality frameworks remains uneven. While accreditation systems tend to regulate communication more explicitly, management standards and policy frameworks often treat communication as a secondary organisational process. This observation indicates a potential gap in the design of quality governance systems, as communication processes play a crucial role in enabling coordination, organisational learning and patient engagement within healthcare institutions.

These findings therefore support the argument that communication should be more explicitly integrated into healthcare quality management systems. Strengthening communication-related standards within quality frameworks may contribute to improving transparency, coordination and organisational learning processes that underpin effective healthcare governance.

## 6. Practical implications

The findings of this study have practical implications for healthcare leaders, quality managers and policymakers responsible for selecting or implementing healthcare quality management systems. In many healthcare systems, organisations may adopt different quality frameworks depending on national regulation, institutional strategy or accreditation requirements. In such contexts, decision-makers benefit from a clearer understanding of how different frameworks structure organisational processes related to quality governance.

The comparative analysis presented in this study suggests that healthcare leaders should consider not only the regulatory structure of quality management systems but also the organisational mechanisms through which these systems address communication processes. The results indicate that accreditation-based frameworks tend to define communication requirements more explicitly, particularly in areas such as patient engagement, interprofessional coordination and complaint management.

For healthcare organisations, these differences may have practical consequences for organisational transparency, patient participation and internal coordination. Frameworks that explicitly incorporate communication-related standards may provide stronger organisational structures supporting teamwork, feedback systems and the systematic management of patient complaints. Such mechanisms may facilitate organisational learning processes and strengthen accountability within healthcare institutions.

Communication is often perceived as a subjective dimension of healthcare quality; however, a growing body of research demonstrates that communication quality significantly influences patient experience, trust and perceived quality of care (Kwame and Petrucka, 2021; Vermeir *et al*., 2015). Emerging evidence suggests that structured communication practices may also contribute to workforce efficiency, interprofessional collaboration and safer care transitions within healthcare organisations (Meneses-La-Riva *et al*., 2025). Consequently, when healthcare organisations have the opportunity to choose between alternative quality management systems, the extent to which communication processes are explicitly addressed within the framework may represent an important consideration for strategic decision-making.

By highlighting structural differences in how communication is integrated into healthcare quality frameworks, the present study provides a practical analytical perspective that may support healthcare leaders in making more informed decisions regarding the adoption and implementation of quality management systems.

## 7. Limitations

Several limitations should be considered when interpreting the findings of this study. The analysis focuses on the conceptual structure of healthcare quality management frameworks rather than their practical implementation within healthcare organisations. Consequently, the study does not capture how communication-related standards are interpreted or operationalised in everyday clinical practice. At the same time, examining frameworks at the system level allows for a clearer understanding of how communication is formally embedded within the institutional architecture of healthcare quality governance.

The study is based on the analysis of publicly available standards and guidance documents describing international healthcare quality frameworks. Although such documents cannot fully reflect the complexities of organisational communication practices in clinical settings, they represent the authoritative sources through which quality management systems define governance mechanisms, organisational requirements and quality improvement structures. Analysing these documents therefore enables a systematic comparison of the formal regulatory logic underlying different quality frameworks.

The comparative analysis also concentrates on a limited number of widely recognised international quality management systems. While this selection does not encompass the full diversity of national or regional quality initiatives, the chosen frameworks represent influential governance models that have shaped international approaches to healthcare quality management. Focusing on these systems makes it possible to compare different institutional logics of quality governance and to identify structural patterns in the way communication is incorporated into quality frameworks.

Finally, the analytical framework developed for this study focuses specifically on communication-related dimensions of healthcare quality management. Such a targeted analytical perspective inevitably simplifies the broader complexity of organisational quality governance systems. However, this focus also represents a methodological strength, as it enables a structured examination of an organisational dimension that often remains implicitly embedded within healthcare quality management frameworks.

## 8. Conclusion

Healthcare quality management systems play a central role in shaping organisational governance, patient safety and accountability within contemporary healthcare systems. As healthcare organisations increasingly rely on structured quality frameworks to guide organisational processes and improvement initiatives, understanding how these frameworks conceptualise key organisational dimensions has become an important area of inquiry.

This study provides a comparative analysis of how communication is incorporated within six internationally recognised healthcare quality management frameworks. Drawing on a structured framework-level analysis, the research examines how communication appears across governance structures, patient engagement mechanisms, team coordination processes and quality improvement systems. In doing so, the study moves beyond the traditional focus on clinical communication and approaches communication as an organisational component of healthcare quality governance.

The results demonstrate that communication is present across all examined quality management systems; however, the level of formalisation varies considerably between governance models. Accreditation-based frameworks tend to regulate communication through explicit standards addressing patient interaction, team coordination and complaint management processes. In contrast, management standards and policy-oriented frameworks integrate communication more indirectly within broader governance and organisational learning structures.

From a scholarly perspective, the study contributes to the literature by introducing a comparative analytical perspective on communication within healthcare quality governance. While previous research has often examined communication in relation to clinical interaction or patient experience, the present study highlights the institutional role of communication within quality management systems. By applying a structured comparative framework analysis, the research demonstrates how communication functions as an organisational infrastructure supporting coordination, accountability and continuous improvement processes within healthcare systems.

The study also contributes methodologically by analysing healthcare quality frameworks at the system level rather than focusing on individual standards or organisational case studies. This approach allows for the identification of structural patterns across different governance models and provides a systematic basis for comparing the institutional logic of healthcare quality management systems.

From a management perspective, the findings underline the importance of considering communication as a strategic organisational dimension of healthcare quality governance. For healthcare leaders responsible for selecting or implementing quality management systems, the explicit integration of communication standards may influence organisational transparency, teamwork and patient engagement mechanisms. As research increasingly emphasises the role of communication in patient safety and organisational learning (Vermeir *et al*., 2015; Ocloo and Matthews, 2016; Wiig *et al*., 2020), recognising communication as a structural component of quality management frameworks may support more effective governance and improvement strategies. Recent healthcare governance literature also suggests that communication-related organisational capabilities may become increasingly important in resilient and patient-centred healthcare systems (Howick *et al*., 2024; Sharkiya, 2023).

Overall, the study demonstrates that examining communication across healthcare quality frameworks provides valuable insights into how quality governance systems structure organisational processes. By highlighting differences in the way communication is institutionalised within quality management models, the research contributes to a more nuanced understanding of the relationship between organisational communication and healthcare quality management.
